# Sight of a Predator Induces a Corticosterone Stress Response and Generates Fear in an Amphibian

**DOI:** 10.1371/journal.pone.0073564

**Published:** 2013-08-29

**Authors:** Edward J. Narayan, John F. Cockrem, Jean-Marc Hero

**Affiliations:** 1 Environmental Futures Centre, School of Environment, Griffith University, Gold Coast Campus, Southport, Australia; 2 Institute of Veterinary, Animal and Biomedical Sciences, Massey University, Palmerston North, New Zealand; Smithsonian's National Zoological Park, United States of America

## Abstract

Amphibians, like other animals, generate corticosterone or cortisol glucocorticoid responses to stimuli perceived to be threatening. It is generally assumed that the corticosterone response of animals to capture and handling reflects the corticosterone response to stimuli such as the sight of a predator that are thought to be natural stressors. Fijian ground frogs ( *Platymantisvitiana* ) are preyed upon by the introduced cane toads ( *Rhinellamarina* ), and we used ground frogs to test the hypothesis that the sight of a predator will induce a corticosterone stress response in an amphibian. Urinary corticosterone metabolite concentrations increased in male ground frogs exposed to the sight of a toad for 1, 3 or 6 h, whereas corticosterone did not change in frogs exposed to another male ground frog, a ball, or when no stimulus was present in the test compartment. The frogs exposed to a toad initially moved towards the stimulus then moved away, whereas frogs exposed to another frog moved towards the test frog and remained closer to the frog than at the start of the test. Tonic immobility (TI) was measured as an index of fearfulness immediately after the test exposure of the frogs to a stimulus. The duration of *TI* was longer in frogs exposed to a toad than to another frog or to a ball. The results provide novel evidence that the sight of a predator can induce a corticosterone response and lead to increased fearfulness in amphibians. In addition, they show that endemic frogs can recognise an introduced predator as a threat.

## Introduction

The hypothalamo-pituitary interrenal (HPI) axis is the central neuroendocrine pathway for physiological stress responses in amphibians. The glucocorticoid hormone corticosterone, the major stress hormone in amphibians (also found in birds, reptiles and many rodents), is secreted by the HPI-axis when individuals respond to a stressor [1–5]. This short-term increase in corticosterone promotes key changes in behaviour and physiology that enable individuals to cope with stress [6–10]. Some of the key behaviours affected by corticosterone in amphibians and also in other vertebrates (small mammals and lizards) include initiation of hiding and defensive behaviours [11–13], increased locomotor activity [14,15], and increased thermoregulatory behaviour [16,17]. The sight of a predator can induce a corticosterone response in birds [18] and exposure to predator odour can induce corticosterone responses in male Sprague–Dawley rats [19]. Thus, corticosterone measures could be used to explore the physiological sensitivity of amphibians to environmental stressors, such as predation and pathogenic infections [20] as well as experimental stressors, such as capture, transportation and toe-clipping [21–23]. Introduced species have had a significant impact on natural ecosystems worldwide, especially through predation [24,25]. Introduced species often cause native animal populations to decline because the native species are oblivious to the possible threat of predation therefore are unable to generate adequate physiological and behavioural responses towards the novel invader [26]. Naturally, prey species should be able to generate physiological and behavioural responses towards a visual predator by increasing stress hormone responses or by hiding, in order to increase its chances of escape and survival [27]. However, some endemic island species are unable to show any such behavioural adjustment due to lack of sufficient evolutionary time in residence with the introduced species [26,28,29]. Currently, there is no empirical evidence to show whether endemic amphibian species are able to recognize novel introduced predatory species as potential physiological stressors.

The Fijian ground frog ( *Platymantisvitiana* ) is currently listed as Endangered (B1ab (v) ver 3.1) by the International Union for Conservation of Nature (IUCN) 2008 (see [30]:). The endemic ground frog population on Viwa Island are preyed upon by the cane toad ( *Rhinellamarina* ) [31–33]. Evidence dating back 20 years highlights that cane toads predate upon froglets and juveniles [34]. The introduced cane toads have also been associated with mortality of native amphibian larvae in Australia [35]. It has also been shown that introduced cane toads have caused rapid alteration of both morphology and physiology of a native Australian species, the red-bellied black snake ( *Pseudechisporphyriacus* ) [36]. In this study, we investigated whether exposure of Fijian ground frogs to a cane toad induced a corticosterone response in the frogs. The degree of fear experienced by an animal when faced by a stimulus that represents a threat is generally correlated with the magnitude of the associated corticosterone response [37], and tests such as tonic immobility (TI) can be used to measure fearfulness in animals [38] including amphibians [39]. We used *TI* tests to measure fearfulness in Fijian ground frogs exposed to the sight of cane toads. We tested the hypothesis that the sight of a cane toad will induce a corticosterone stress response and fearfulness in the Fijian ground frog. Furthermore, we also investigated whether the presence of cane toads altered the movement of the ground frogs to provide an insight into the potential effects of toads on the locomotory behaviour of ground frogs.

## Materials and Methods

### (a) Ethics statement

All experiments were conducted according to relevant national and international guidelines. Ethical clearances were obtained from the Department of Environment, Fiji Islands for the collection of the endangered Fijian ground frogs on Viwa Island (18^o^00´S, 175^o^00´E), a small (60 ha) island located 900 m off the coast of mainland Viti Levu, Fiji. The traditional owner of the land (Chief of Viwa Island; Ratu Dovi Komaisavai) permitted us to undertake this research on Viwa through a traditional ceremony that was conducted prior to this study. The Griffith University Animal Ethics Committee (ENV/13/11/AEC) that approved the interventions with the amphibians in this study. All frogs were released back on into their natural habitat upon completion of the study in accordance with the Australian health check standards for amphibians [40].

### (b) Field sampling and captive housing

We used adult male Fijian ground frogs for this study because of their easy accessibility compared to female ground frogs, and to reduce any confounding effects of reproductive condition on subsequent stress and behaviour measurements. Ground frogs and cane toads were caught at night (2000-2200h) on Viwa Island within shared terrestrial habitats in December 2011 during the annual breeding season of the ground frogs. Adult male frogs were identified by their distinct ‘stress call’ upon capture, as described earlier [41] while adult male cane toads were identified based on morphological characteristics [42] and a rough dorsal surface. Adult male ground frogs weighed 33.5 + 2.5 g, and had mean snout-vent length (SVL) of 35 mm (*n* = 112; 91 experimental frogs and 21 test stimulus frogs). Adult male cane toads weighed 55.0 + 1.5 g, and had mean SVL of 65 mm (*n* = 21 test stimulus toads). We used a urinary corticosterone metabolite enzyme-immunoassay (EIA) to measure urinary corticosterone metabolite concentrations (referred to hereafter as urinary corticosterone concentrations) using our established protocols for anuran amphibians [21,43–46]. Urine samples were collected from individual frogs on the night of capture on Viwa Island. For urine sampling, each frog was gently held above a sterile plastic cup (diameter = 100 mm) and its underbelly abdomen was briefly massaged to promote urination, which occurred always within 1 min. The volume of urine excreted by each frog ranged from 200 µL -3 mL. Labelled urine samples were kept in a freezer prior to assay (no longer than 1 month). Frogs and toads were transported overnight from Viwa Island to a nearby field station (within 5 km from Viwa Island) on mainland Viti Levu, where the experiments were conducted. At the field station, the frogs were housed individually in plastic “home containers” with meshed lids (10 x 35 x 10 cm). Frogs were provided with food (house crickets, *Achetus* *domesticus* ) every second day and distilled water was sprinkled twice daily into the home container to keep it moist. The arena containing the home containers had a similar photoperiod to Viwa Island. Frogs were adjusted to captivity for 9 days before the experiments began. This adjustment period was selected based on our earlier results on the physiological adjustment of amphibians to captivity [43,45]. Urine samples were collected from each frog after 0.5, 5 and 9 days to ensure that corticosterone concentrations had returned after 9 days to concentrations similar to the time of capture concentrations.

### (c) Experimental design

The responses of adult male ground frogs to three experimental treatments were examined 10 days after capture on Viwa Island. Individual frogs were introduced to either a cane toad, another adult male ground frog or to a ball, or not exposed to any stimulus. The home container, which was also used as the experimental chamber, was divided in half using plastic wire mesh (15 mm x 15 mm) through which frogs could perceive (see and smell) the test stimulus. This was confirmed by placing a live or a dead house cricket in the test compartment and noticing the frog’s movement in the test chamber.

Male ground frogs (n = 21) and toads (n = 21) that were used as test stimuli were caught on Viwa Island on the same night in December 2011 as the experimental frogs, and were acclimated in separate home containers away from the arena where the experimental frogs were kept. On the day of the experiment, male frogs that were used as controls (n = 28) were sampled for urine 0, 1, 3 or 6 h (n = 7 frogs per time period) without any stimulus being present in the test compartment. Experimental male ground frogs (n = 63 frogs) were divided into groups and subjected to one of the three treatments (cane toad, another adult male ground frog or a ball), with a single urine sample collected 1, 3 or 6 h (n = 7 frogs sampled per time period for each treatment) after the experiment began. At the start of each experimental session a piece of black cardboard was placed in the middle of the test chamber and then a toad, frog or the ball was placed into the test compartment by the observer. The cardboard was removed so that the male ground frog could then see the test stimulus. The control frogs were also exposed to the placement and then urine was collected upon removal of the cardboard divider. Sampling time periods of 1, 3 or 6 h were used based on our previous works that showed that physiological stress responses of anurans (e.g. ground frogs) appeared in urinary corticosterone metabolites within 6 h after exposure to experimental stressor, such as adrenocorticotropic hormone (ACTH) injection [44].

### (d) Behaviour

A rectangular sheet of white paper with lines drawn with a permanent marker across the sheet at 20, 40, 60 and 80% of the length of the area of each home container in which the ground frog was present was placed beneath the home container before the tests began so the observer could see the lines from a standard distance of 0.5 m. The lines divided the area in which the male frog was present in five locations which were termed 1, 2, 3, 4 and 5, with location 1 closest to the test compartment and location 5 furthest away from the test compartment at the start of the treatment. The location of the frog was recorded at the start of each test (0 min location). The location was then recorded 5, 15, 20, 40 and 60 min after the test began for frogs sampled for urine at 1 h; or 5, 15, 20, 40, 60, 120, 180 min after the test began for frogs sampled for urine at 3 h; or 5, 15, 20, 40, 60, 120, 180, 240 and 360 min after the test began for frogs sampled for urine at 6 h. Each frog was picked up for urine collection immediately after the final location observation was made for each treatment.

The duration of tonic immobility (*TI*, also known as the immobility reaction [47]; was measured after urine was collected from each frog. The tonic immobility test is a quantitative measure of fear that has been used in previous amphibian studies [47,48]. Each frog was removed from its home container and placed on its back on the test surface (flat plastic board 15 x 15 x 10 mm) and a timer was started immediately. The frog was gently held by the hands until the hands could be moved away and the frog remained on its back, following the procedure of [39]. The latency time (Sec) was the time when the hands could be moved away while the frog remained on its back. The time taken for the frog to upright was recorded as the duration of immobility. Frogs were returned into their home containers at the end of the test and returned to Viwa Island at the end of the experiment.

### (e) Corticosterone enzyme-immunoassay

A urinary corticosterone enzyme-immunoassay (EIA) that was previously validated for measuring conjugated corticosterone metabolites in frog urine was used for this study [21,43–45]. Urinary corticosterone metabolite concentrations in frog urine were determined using a polyclonal anti-corticosterone antiserum (CJM06, UC, Davis California) diluted 1: 45 000, horseradish peroxidise-conjugated corticosterone label diluted 1: 120 000 and corticosterone standards (1.56–400 pgwell^-1^). Cross reactivity of the CJM06 anti-corticosterone antiserum was reported earlier as 100% with corticosterone, 14.25% with desoxycorticosterone and 0.9% with tetrahydrocorticosterone [44]. Intra- (within) and inter- (between) assay coefficients of variation were determined from high- (~70%) and low- (~30%) binding internal controls run on all assays. Intra assay CVs were 3.9% and 4.4% for low, and high percentage bound controls, and inter-assay CVs were 4.2% and 6.8% for low, and high percentage bound-controls respectively. Assay sensitivity was calculated as the value 2 standard deviations from the mean response of the blank (zero binding) samples. Mean assay sensitivity for the corticosterone EIA was 1.16 + 0.27 pg/well (n = 24). Creatinine (Cr) was measured in each frog urine sample as an index of the urinary corticosterone metabolite concentrations because it is excreted at a constant rate and is therefore a good index of the amount of time over which hormones have been metabolized into the urine regardless of the volume of the sample [44]. Urinary corticosterone metabolite concentrations were expressed in relation to urinary creatinine concentrations as pg corticosterone metabolites/ug Cr.

### (f) Statistical analysis

Statistical analyses were performed using Prism (GraphPad Software Inc.). Urinary corticosterone metabolite concentrations were transformed to logarithms, and D’Agostino & Pearson omnibus test confirmed normality and homogeneity of variances for log-transformed data. Urinary corticosterone metabolite concentrations in adult male ground frogs after they were captured from the wild and transferred into captivity were compared between days using one-way Repeated Measures ANOVA. Post-hoc comparisons were done between times (days 0, 0.5, 5 and 9) using Bonferroni’s Multiple Comparison Test.

All of the physiological and behavioural data (urinary corticosterone metabolite concentrations, mean locations of frogs in the test compartment and mean tonic immobility scores) at times 1, 3 or 6 h since the start of each treatment were compared between groups (control and treatments) using two-way ANOVA followed by post hoc comparisons.

Comparisons of the physiological and behavioural data between times (1, 3 or 6 h) within each frog group (control and treatments) were done using two-way ANOVA followed by post-hoc comparisons. Data are presented as individual points or mean + S.E.

## Results

### (a) Urinary corticosterone after capture

**Figure 1:**
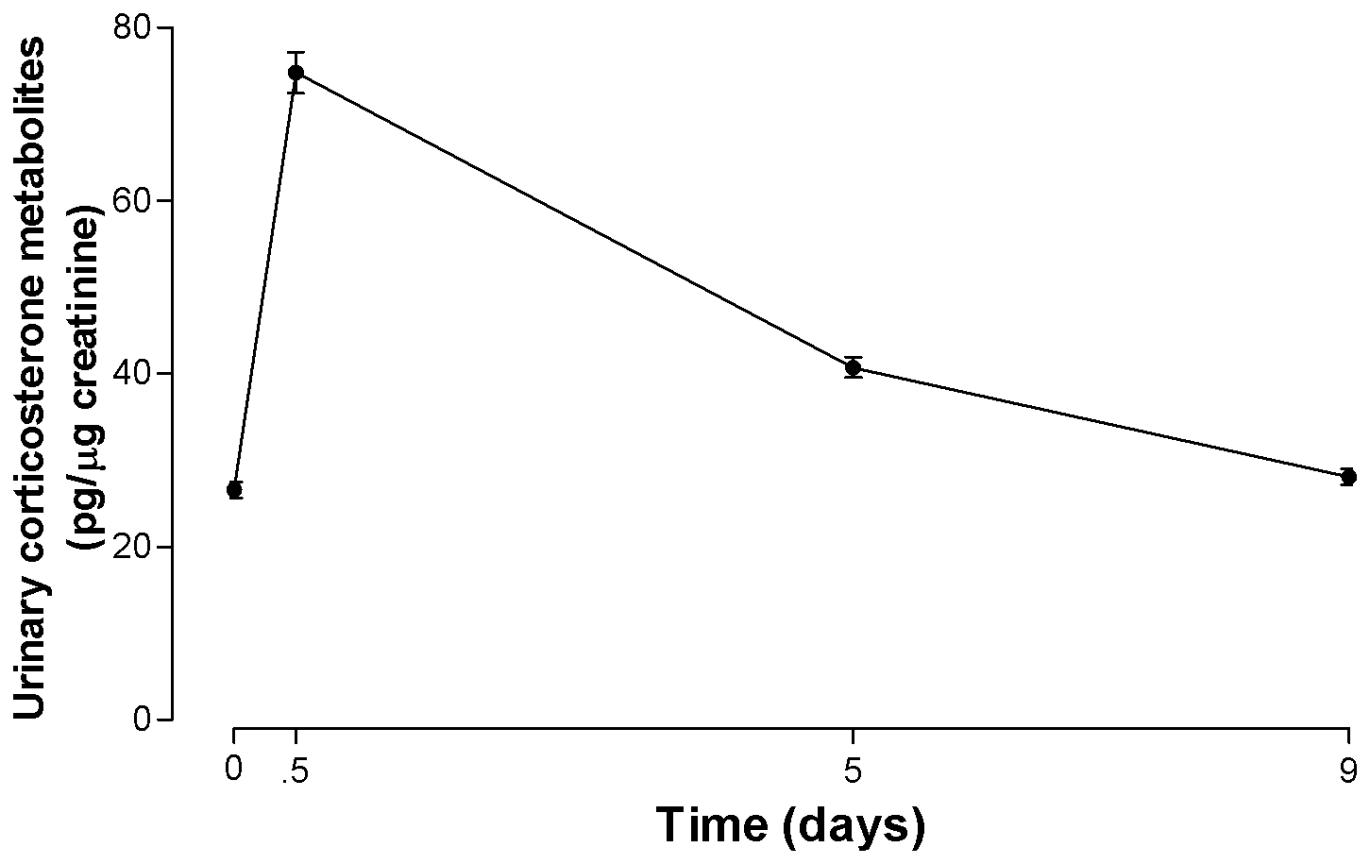
Mean (+ S.E) urinary corticosterone metabolite concentrations at capture (0 days) and after 0.5, 5 and 9 days in captivity in 91 male Fijian ground frogs.

Urinary corticosterone concentrations in all male frogs (n = 91) had increased from 0.5 days after capture (Figure 1). Corticosterone decreased between 0.5 and 5 days and, in most frogs, decreased further from 5 to 9 days after capture (Figure 1). Mean urinary corticosterone concentrations in the frogs changed significantly after capture (One-way ANOVA, *F*_3,273_ = 216.8, *p* < 0.0001, Figure 1). The mean urinary corticosterone concentration increased between the time of capture and day 0.5 (*p* < 0.05), decreased on day 5 (*p* < 0.05), and after 9 days in captivity did not differ from initial concentrations at the time of capture (*p* > 0.05).

### (b) Urinary corticosterone responses to test stimuli

**Figure 2:**
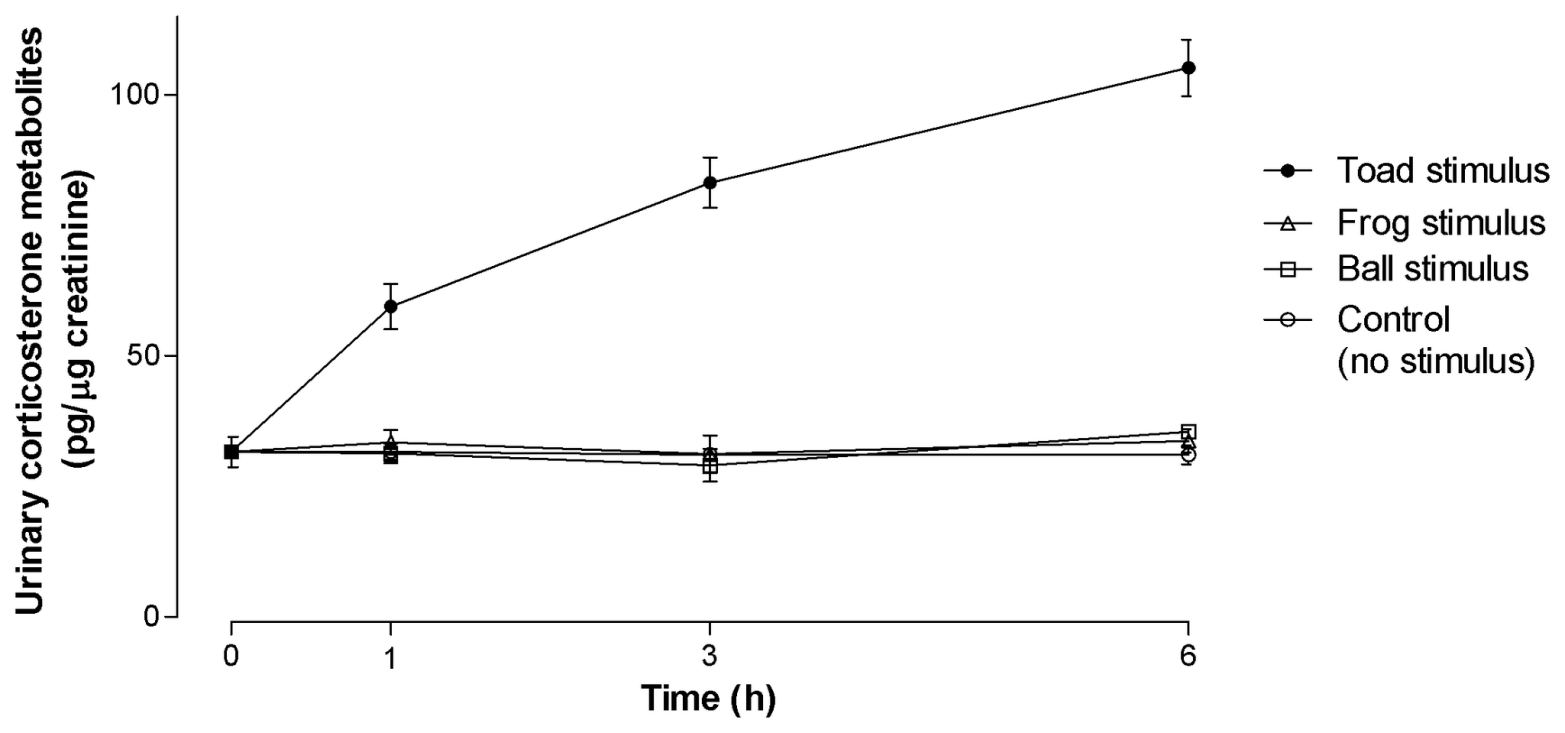
Mean (+ S.E.) urinary corticosterone metabolite concentrations in male Fijian ground frogs during exposure to a cane toad, a ball, another male ground frog or control (no stimulus). Sample sizes at each time point were n = 7. Mean urinary corticosterone data for each four group (control and three treatments) were used to obtain the data for time = 0 h.

A two-way ANOVA of mean urinary corticosterone metabolite concentration at 1, 3, or 6 h after exposure to a test stimulus showed that corticosterone metabolite concentrations varied with time (*F*_3,96_ = 29.24, *p* < 0.001) and treatment (*F*_3,96_ = 153.7, *p* < 0.001), with a significant interaction between time and treatment (*F*_9,96_ = 26.04, *p* < 0.001). There were significant differences in mean urinary corticosterone metabolite concentrations between frogs in the control group and frogs that saw the cane toad 1, 3 and 6 h after exposure to the sight of the toad (*p* < 0.001; Figure 2). There were no significant differences in mean urinary corticosterone metabolite concentrations at these times between frogs that saw another frog or a ball compared with the control group (*p* > 0.05 for all comparisons; Figure 2). The mean urinary corticosterone concentrations were significantly different at times (1, 3 or 6 h) for frogs that saw the toad compared to those that saw the ball or another frog (*p* < 0.05 for all comparisons). Mean urinary corticosterone metabolite concentrations increased in frogs that saw a toad and were significantly higher compared with initial 0 h corticosterone concentrations at times 1, 3 and 6 h (*p* < 0.001; Figure 2). The highest mean urinary corticosterone metabolite concentration was at 6 h (105.14 + 5.4 pg/µg Cr) for the frogs that were exposed to the cane toad. Corticosterone did not increase from initial concentrations in frogs that saw another frog or a ball or for the control group (Figure 2).

### (c) Behaviour

**Figure 3:**
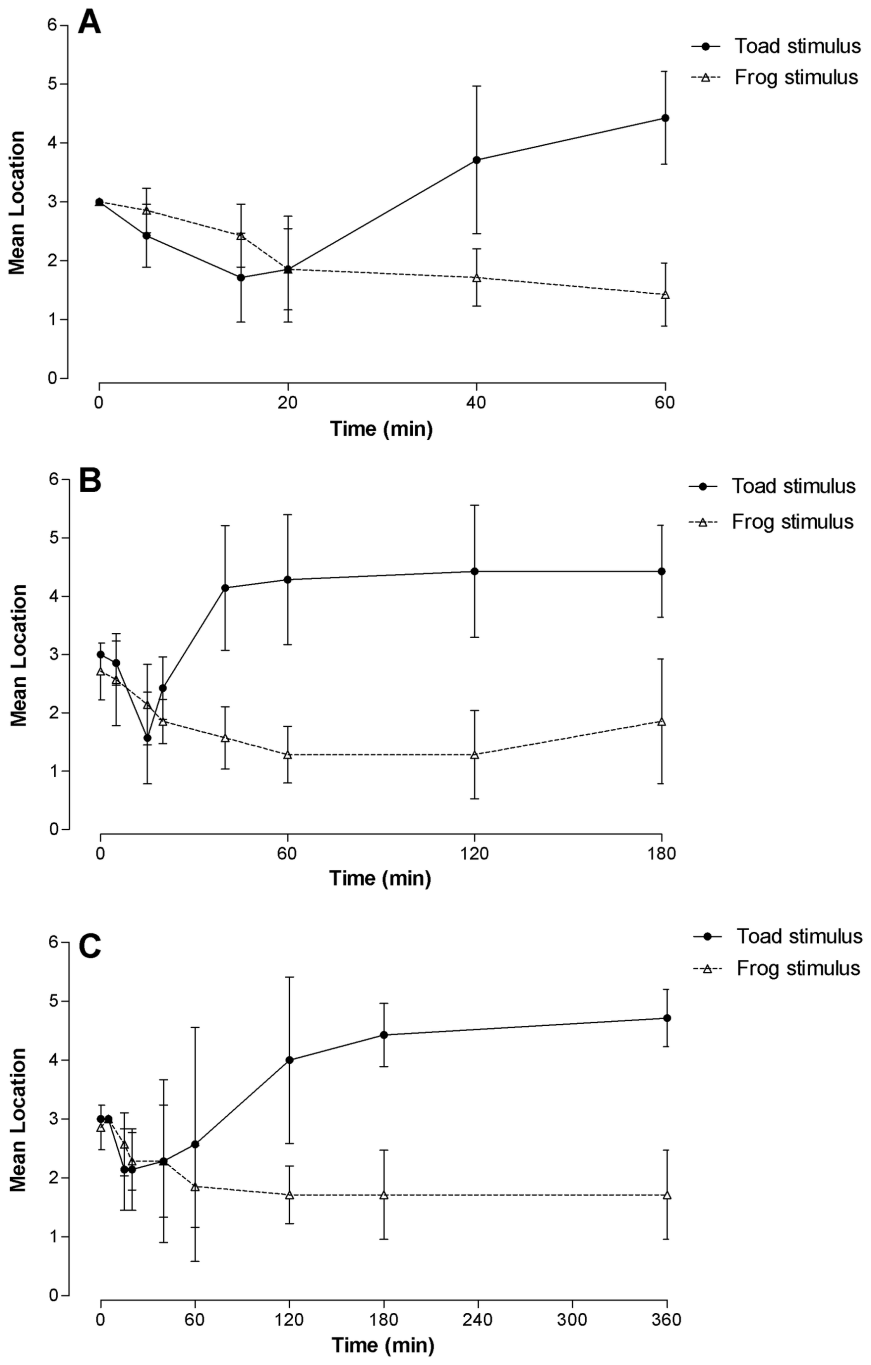
Mean (+ S.E.) locations of the Fijian ground frogs during exposure to a cane toad or another frog. Location 1 was closest to the test compartment and location 6 was furthest away from the test compartment. The location of the frog was recorded at 5, 15, 20, 40 and 60 min after the test began (Fig. 3A, frogs sampled up to 60 min) or 5, 20, 40, 60, 120, 180 (Fig. 3B, frogs sampled up to 180 min) or 5, 20, 40, 60, 120, 180, 240 and 360 min (Fig. 3C, frogs sampled up to 360 min). Sample sizes at each time point were n = 7.

Adult male ground frogs were usually sitting in the middle of their home container (location number 3) before being subjected to the test stimulus. The locations of the ground frogs changed when the frogs were exposed to some of the test stimuli. Frogs exposed to cane toads moved towards the test compartment within the first 15-20 min and then moved away and stayed as far away as possible for the remainder of the test period (Figure 3). Frogs exposed to a male ground frog in the test compartment moved towards the compartment within the first 15-20 min and stayed close to the test compartment throughout the remainder of the test period (Figure 3). There was little or no change in the locations of the ground frogs in the control group and in the presence of a ball. There was a significant effect of test stimulus (*F*_3,96_ = 94.98, *p* < 0.0001) and time (*F*_3,96_ = 3.22, *p* = 0.026) on the location of the male ground frogs, and a significant interaction between test stimulus and time (*F*_9,96_ = 11.39, *p* < 0.0001). There was a significant difference in locations of frogs over 1, 3 or 6 h between frogs subjected to the toad or another frog (*p* < 0.05; Figure 3A–C). There was no significant difference in locations of the frogs between those subjected to a ball or a control group (*p* > 0.05).

**Figure 4:**
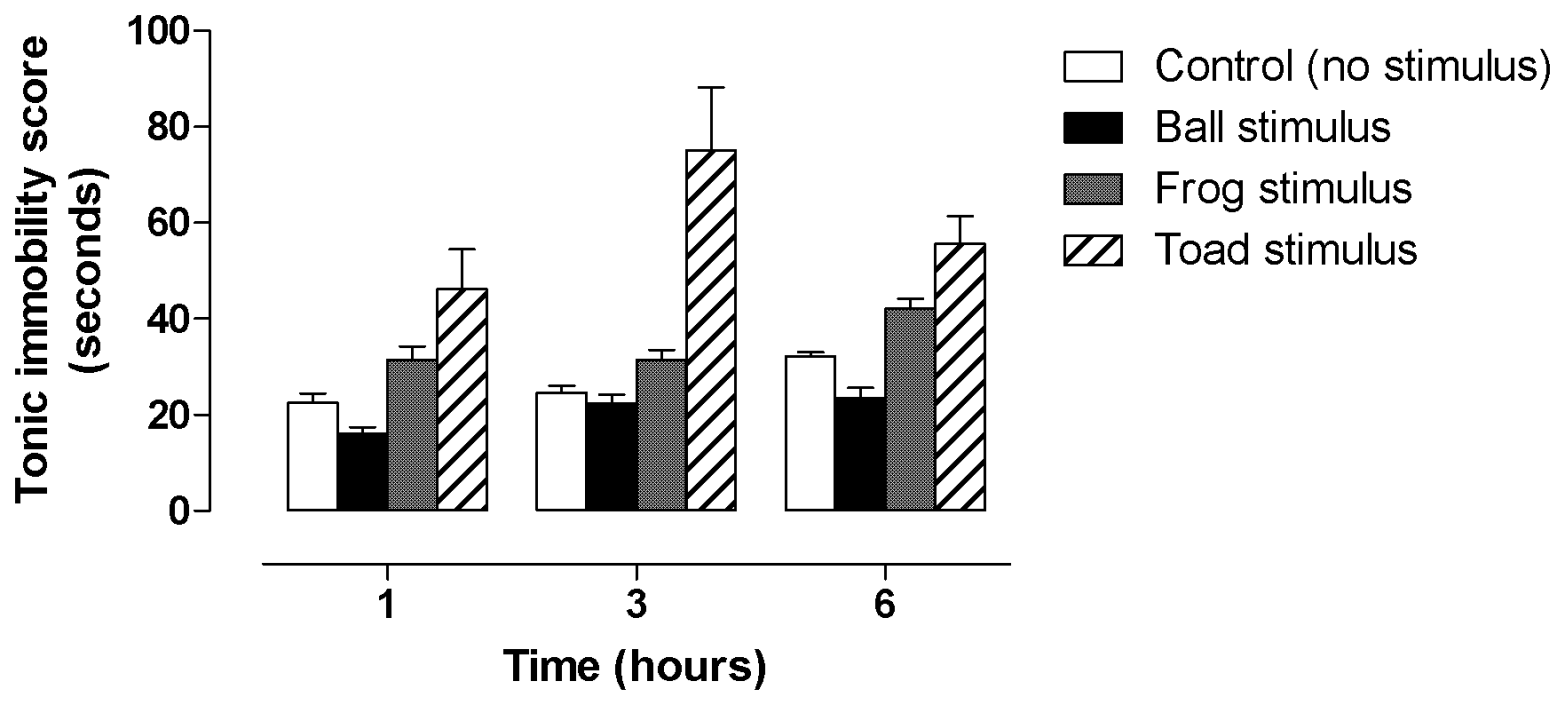
Histogram showing mean durations of tonic immobility (seconds) of male Fijian ground frogs after exposure to a cane toad, a ball, another frog or no stimulus. Sample sizes at each time point were n = 7.

The mean duration of tonic immobility (TI) of the male ground frogs differed significantly between stimuli (*F*_3,72_ = 56.09, *p* < 0.0001, Figure 4) and time (*F*_3,72_ = 11.78, *p* < 0.0001, Figure 4). There was no significant interaction between treatment and time (*F*_3,72_ = 1.97, *p* = 0.080, Figure 4). The frogs that were subjected to a toad had the longest durations of *TI* at 1, 3 and 6 h (Figure 4). The highest mean *TI* duration (75.0 + 13.2 seconds) was recorded at 3 h for the frog group that was subjected to the sight of a toad. There was no significant difference between time in mean *TI* durations for frogs that saw a frog or ball (Figure 4; *p* > 0.05 for all time comparisons). There was a significant difference between the times in mean *TI* durations of the control frogs and frogs that had perceived the cane toad (Figure 4; *p* < 0.05 for all time comparisons). The mean *TI* durations of the frogs that saw another frog were significantly shorter than those of frogs that saw a toad, and significantly longer than the mean *TI* durations of frogs that saw a ball 1, 3 or 6 h (Figure 4; *p* < 0.05).

## Discussion

This is the first study to show that amphibians have a corticosterone response to the sight of an invasive predator. Frogs that saw the predator also had greater fearfulness, as measured by the duration of tonic immobility (TI) immediately after exposure to the predator, than frogs that saw another frog or saw a ball. These results show that the Fijian ground frogs perceived the sight of a cane toad to be a threat, and experienced fearfulness at the same time as their hypothalamo-pituitary interrenal (HPI) axis was activated and corticosterone secretion increased.

The adult male ground frogs experienced greater fearfulness, as measured by higher durations of *TI* in the presence of cane toads than in the presence of another frog or a novel object (ball). Tonic immobility has been demonstrated in a wide range of animals, including birds, fish, rabbits and guinea pigs, to reduce the probability of continued attacks by an intruder [49]. Experimental elevation of corticosterone has also been shown to increase the duration of immobility in birds [50]. Furthermore, the male ground frogs altered their behaviour (moved further away from the test chamber) following visual detection of the cane toad in the experimental chamber. Ground frogs are often found in areas of minimal cover, such as open-exposed bare ground and exposed perches on tree trunks at night-time [51]. Cane toad encounters with ground frogs are not uncommon and the frogs usually evade the toads by retreating into terrestrial substrates, such as leaf litter, or moving up into the vegetation [41]. In their study [52], demonstrated that behaviour towards any biological stressor (such a predator) could differ between frog species depending on micro-habitat preferences. For example, more terrestrial dwelling northern leopard frogs ( *Rana* *pipiens* ) escaped towards land while their aquatic dwelling congener, the green frog ( *R* *. clamitans* ), escaped towards the water when approached by snakes or humans around natural ponds. Seeking refuge following a stressful encounter could be beneficial if the intruder is a visual hunter, but may prove detrimental when the predator is able to enter these substrates. There is empirical evidence that the spatial distributions of the cane toads and Fijian ground frogs on Viwa Island overlap quite significantly [31]. We studied the male ground frogs during breeding thus future studies should investigate whether responses (fearfulness and corticosterone responses) of male frogs are similar outside the breeding season, including their correlation with testosterone [53,54] and body condition [7]. Similar testing could be done on female frogs to test if physiological responsiveness to the cane toad is affected by sex, condition and reproductive stage [55].

Behavioural and physiological stress responses are under some level of genetic control [56], and responsiveness could increase from natural selection pressures, such as that imposed by predation [57]. We have shown that ground frogs perceive cane toads as a threat and move away from toads. On Viwa Island other potential predators of ground frogs, such as the introduced small Indian mongoose ( *Herpestes* *javanicus* ) have never been present, while rats ( *Rattus* *spp.* ) and feral cats (*Felis catus*) have already been eradicated from the island [31]. It could be that there has been selection pressure in the frogs to recognise toads as predators, which is also not known. In future, it would be worthwhile to investigate whether the ground frogs are able to show similar physiological and behavioural responses in the presence of potential native predators such snakes ( *Candoia* *sp.* ) as well as in the presence of invasive species (such as rats and feral cats). Certain native species that have evolved in the absence of any intruders could also display specific behaviours in response to the intruder. A classic example was an earlier study by [58] in which the marine iguana ( *Amblyrhynchus* *cristatus* ) that has been isolated from mainland populations for over 15 million years was tested for behavioural and physiological stress hormone responses to some novel predators. Their study demonstrated that the ‘naïve’ marine iguana were able to generate anti-predator responses (both stress hormonal and behavioural responses) in the presence of novel predators (dogs and cats) [58]. Both this study and another by [36] emphasized that physiological stress and behavioural responses of native species against novel introduced species demonstrate cases of rapid evolution under the influence of strong predatory selection pressure. Earlier [18], found a marked increase in plasma corticosterone in captive great tits ( *Parus* *major* ) that saw an owl, which is a natural predator. In another study [59], studied the stress hormone responses of free-living male pied flycatchers ( *Ficedula* *hypoleuca* ) that were exposed for 10 min to a stuffed weasel ( *Mustela* *sp.* ) model, a stuffed great spotted woodpecker ( *Dendrocopos* *major* ), or a stuffed flycatcher that was placed on or near their nest box to act as a territorial intruder. Exposure to a weasel or to a territorial intruder elevated plasma corticosterone concentrations in flycatchers caught 10-25 min after the beginning of a test stimulus.

Overall, this study has provided novel evidence that amphibians can show a corticosterone response to the sight of a predator. Determining whether the physiological stress and behavioural responses of ground frogs towards cane toads are a result of natural selection or learnt responses could be tested by replicating our experimental protocols with a naïve population of ground frogs from neighbouring Gau Island (located at 18.00° S and 179.30 ° E), where there are currently no cane toads present [2,60].
